# Infection rate among close contacts of patients with coronavirus disease in Japan: a descriptive study and literature review

**DOI:** 10.2478/abm-2023-0051

**Published:** 2023-10-09

**Authors:** Osamu Yamamura, Hidenori Onishi, Ippei Sakamaki, Ryousuke Fujita, Hirofumi Miyashita, Hiromichi Iwasaki

**Affiliations:** 1 Department of Community Medicine, Faculty of Medical Sciences, University of Fukui, Fukui 910-1193, Japan; 2 Department of Infectious Diseases, Faculty of Medical Sciences, University of Fukui, Fukui 910-1193, Japan; 3 Department of Biostatistics, Faculty of Medical Science, University of Fukui, Fukui 910-1193, Japan; 4 Department of Health and Welfare, Fukui Prefecture, Fukui 910-8580, Japan; 5 Department of Infection Prevention and Control, University of Fukui Hospital, Fukui 910-1193, Japan

**Keywords:** close contact infectious disease transmission, contact time, COVID-19, protective measures, social distancing

## Abstract

**Background:**

In Japan, on April 20, 2020, the definition of a close contact regarding coronavirus disease (COVID-19) was changed from a long-term contact time to a specified contact time of 15 min and from a contact distance of 2 m to 1 m.

**Objectives:**

We aimed to determine the severe acute respiratory syndrome coronavirus 2 (SARS-CoV-2) infection rate among close contacts of patients with COVID-19 and determine the impact of the infection on transmission among close contacts.

**Methods:**

The numbers of SARS-CoV-2 tests, SARS-CoV-2-positive cases, and close contacts of patients with COVID-19 were assessed between March 2020 and February 2021 in Fukui Prefecture, Japan. The study period was subdivided into 3 periods. The second and third period contained data with the changed definition of close contact.

**Results:**

Overall, 32,238 SARS-CoV-2 tests were performed. There were 545 patients with COVID-19 and 1487 close contacts, of whom 267 tested positive. The highest infection rate occurred in period 3. Distance, protective measures, and contact time with COVID-19 patients influenced the increased infection rate. The infection rate showed a rising trend from 11.1% in period 1 to 19.2% and 20.0% in periods 2 and 3, respectively (Cochran–Armitage test; *P* < 0.004). Multivariate analysis revealed that female sex was an independent risk factor for infection of close contacts (odds ratio: 2.23; 95% confidence interval: 1.700–2.930).

**Conclusions:**

Female sex is a risk factor for transmission by close contacts. The rate of infection among close contacts may be associated with contact time, contact distance, and protective measures.

The first case of coronavirus disease (COVID-19) in Japan was confirmed in January 2020 [1]. The number of COVID-19 cases has been fewer, and restrictive measures have been less stringent than those in other countries [1]. Additionally, fewer diagnostic tests have been performed than those in some other countries, focusing on clusters, which may have led to the underestimation of the total number of COVID-19 cases [1]. Since the start of the outbreak in Japan, there have been second, third, fourth, fifth, sixth, seventh, and eighth peaks in early August 2020, early January 2021, mid-May 2021, late August 2021, early February 2022, early August 2022, and mid-August 2022, respectively [1, 2]. As of March 2023, about 419,640 polymerase chain reaction (PCR) tests were performed per day in Japan for severe acute respiratory syndrome coronavirus 2 (SARS-CoV-2) [3].

Globally, the cumulative number of COVID-19 cases exceeded 759 million, while the number of cases recorded in Japan exceeded 33.16 million as of March 2023, and the situation remains critical to date [2]. Social distancing is important in preventing COVID-19; therefore, maintaining a distance of at least 2 m from other people and wearing face masks are considered effective preventive measures [4]. Close contact with an infected person is a major route of SARS-CoV-2 infection; thus, countermeasures targeting close contact with people confirmed to have COVID-19 are required. The SARS-CoV-2 infection rate among close contacts of infected persons has been reported as 0.8% in Taiwan and 3.7% in China (Guangzhou) [5, 6]. Moreover, the SARS-CoV-2 attack rate among close contacts has been reported to be 0.7% in Taiwan, 3.5% and 4.4% in Guangzhou (China), 6.5% in Hubei Province (China), and 15% in Japan (Nagano Prefecture) [5,6,7,8,9,10]. Reports of secondary infections among close contacts include infection and attack rates.

Preventing the spread of COVID-19 among close contacts is important to avoid the collapse of the medical practice. In Japan, the definition of close contacts was changed on April 20, 2020 [11]. The following 2 changes were made: (1) The beginning of the day of contact with a person with COVID-19 changed from “the day of onset of illness” to “2 days before the day of onset of illness” [11, 12]; and (2) the standard for determining close contact was changed from “contact within 2 m” to “contact within 1 m for at least 15 min” [11, 12]. In this study, we aimed to determine the SARS-CoV-2 infection rate among close contacts of patients with COVID-19 and to determine the impact of the infection on the transmission among close contacts. In addition, a brief literature review was conducted on the transmission of the disease among close contacts.

## Methods

A COVID-19 survey was carried out in Fukui Prefecture, Japan, as part of an outbreak investigation based on the Act on the Prevention of Infectious Diseases and Medical Care for Patients with Infectious Diseases in Japan. Fukui Prefecture has a population of 761,306 people. All data were anonymized before analysis, and only researchers appointed by the Fukui Prefectural Government were authorized to access, analyze, and use the COVID-19 survey data.

This study was conducted with the approval of the University of Fukui Medical Research Ethics Review Committee (certificate of approval No. 20210037). The requirement for informed consent was waived because the study was based on a retrospective analysis of anonymized data. All researchers involved in this study complied with Ethical Guidelines for Medical and Biological Research Involving Human Subjects (MEXT/MHLW/METI Notification No. 1 of March 23, 2021).

### Definition of COVID-19-positive cases and close contacts

A confirmed COVID-19 case was defined when a person tested positive for SARS-CoV-2 by real-time reverse-transcription PCR [13]. Close contact was defined according to the Active Epidemiological Investigation in Patients with Novel Coronavirus Infection guidelines of the National Institute of Infectious Diseases [11, 12]. Close contact was considered infected based on a positive PCR test result within 14 days of the last contact with a COVID-19 patient. The infection rate was calculated as the percentage of close contacts who became COVID-19-positive within 14 days $\left(\text{Infection}\;\text{rate}=\frac{\text{Persons}\;\text{infected}\;\text{through}\;\text{close}\;\text{contact}}{\text{Close}\;\text{contact}}\times 100\right).$ . Close contacts were identified at the health center.

### Parameters analysis

This study was retrospective and observational. We used existing data from COVID-19 surveys conducted in Fukui Prefecture, Japan, to examine the numbers of COVID-19 tests, COVID-19-positive cases, and close contacts of patients with COVID-19 between March 2020 and February 2021. Based on the increase and decrease in the number of COVID-19 cases, the study period was subdivided into 3: period 1 was from March to June 2020, period 2 was from July to September 2020, and period 3 was from October 2020 to February 2021. According to these 3 periods, we compared the number of SARS-CoV-2 tests, number of infected cases, ages of the infected patients, presence or absence of underlying disease, number of close contacts of patients with COVID-19, and changes in the infection rate among close contacts. The infection rate was evaluated based on various countries’ different definitions of close contacts (Taiwan, Singapore, China, and Japan). For the article search, we used PubMed and selected articles in which the “definitions of close contact,” “infection rate,” and “attack rate” were described in English and considered mainly Asian countries. This study presents the infection rate among close contacts.

### Definition of close contacts in Japan

“Close contact” is someone who has been in contact with an infected person (tested positive for COVID-19 via PCR testing) and meeting the following conditions [12]: (1) a person who lives with a COVID-19 patient [12]; (2) a person who had long-term contact with a patient (e.g., a person who was sitting front and back 2 rows in an international flight or within 2 m in a domestic flight) [12]; (3) a person who has examined, nursed, or cared for a patient without appropriate infection; (4) a person who may have come into direct contact with contaminants, such as airway secretions or bodily fluids [12]; (5) a person who has been in contact with a patient for >15 min without necessary infection prevention measures within touching distance (1 m) [12]. The contact period starts from 2 days before the onset of symptoms or specimen collection until the end of isolation. Based on these conditions, the public health center conducts interviews and makes a comprehensive judgment [12].

The definition of close contacts changed after April 20, 2020 [11]. The following 2 main changes were made: (1) The beginning of the day of contact with a person confirmed to have COVID-19 changed from “the day of onset of illness” to “2 days before the day of onset of illness” [11], and (2) the standard for determining close contact changed from “contact within 2 m” to “contact within 1 m for at least 15 min.” We speculate that the infection rate increased in the second and third periods than in the first period because of the change in definition [11].

### Contact tracing in Japan

Contact tracing in Japan, as in other countries, is “prospective,” which starts with newly confirmed infectious cases; identifies close contacts; and searches for future infectious cases [14]. In Japan, epidemiological investigations were conducted aggressively to identify the source of cluster infections (outbreaks) and promptly implement countermeasures to minimize the spread of infection. Cluster infection control involves retrospectively determining common sources of infection when infected persons are identified. The concept of 3Cs (closed spaces, crowded places, and close contact settings) that are common to all sources of infection was postulated [14].

### Definition of close contacts in each country

**Table 1** summarizes the definitions of close contact in Taiwan, Singapore, China (Guangzhou and Hubei Province), and Japan (Fukui and Nagano Prefectures) involving contact duration, contact distance, and protective measures [5,6,7,8,9,10].

**Table 1.** Characteristics of definitions of close contacts and infection and attack rates in four Asian countries.

	**Time**	**Distance**		Protective measure^*^	**Observation period**	**Secondary infection rate**	**Secondary attack rate**

		**<2.0 m**	**<1.0 m**				
Taiwan	15 min or more (conversing)	○ (medical institution)		None (medical institution)	Jan – Apr 2020	0.8%	0.7%
Singapore	30 min (with patient)	○		-	Jan – Apr 2020	-	2.4%
Guangzhou, China			○	None (social life)	Jan – Mar 2020	3.7%	3.5%
Guangzhou, China			○	None (social life)	Before March 2020	-	4.4%
Hubei Province, China			○	None (social life)	Jan – Feb 2020	-	6.5%
Nagano, Japan	15 min or more (contact)		○	None (social life)	Aug – Sep 2020	-	15.0%
Our study	Had prolonged contact	○		None (social life)	Mar – Jun 2020	Period 1:13.3%	-
Fukui, Japan	15 minutes or more (contact)		○	None (social life)	Jul – Sep 2020 Oct 2020 – Feb 2021	Period 2:19.2% Period 3:20.0%	- -

Other conditions (which varied from country to country) included living together (in the same room), engaging in medical and welfare services, diet, vehicles, number of days of symptom onset in COVID-19 index patients, and contact with infectious agents.

* Protective measure (which varies from country to country) included personal protective equipment (PPE) (mask, face shield, goggles, gown, gloves), etc.

### Statistical analyses

Age is presented as median and interquartile range. Categorical variables are presented as frequencies and percentages. Two- and four-group comparisons were performed using EZR (version 1.41; Saitama Medical Center, Jichi Medical University, Saitama, Japan), a graphical user interface for R (The R Foundation for Statistical Computing, Vienna, Austria) [15]. The Mann–Whitney *U* test and chi-square test (using Yates’ continuity correction) were used for paired group comparisons. Fisher's exact test (using the Holm adjustment for multiple comparisons) and the Kruskal–Wallis test (using the Holm adjustment for multiple comparisons and post-hoc analysis) were used for 3-group comparisons. The Cochran–Armitage test, a test of trend, was used to evaluate the increasing/decreasing trend in the percentage of infections among the close contacts in the first, second, and third periods. To identify the risk factors associated with infection by close contacts, we also performed a multiple logistic regression analysis (binomial logistic regression analysis) with the presence or absence of infection by the close contact. BellCurve for Excel (version 3.20; Social Survey Research Information Co. Ltd., Tokyo, Japan) was used to analyze the stratified categorical variables. The Breslow–Day test was used to assess the homogeneity and uniformity of the stratified odds ratios (ORs). The Cochran–Mantel–Haenszel test was used to test for conditional independence. The Cochran–Mantel–Haenszel test is a test of overall independence adjusted for the effects of stratification factors (period factors) and an estimation of the overall OR adjusted for the effects of stratification factors (period factors) in data with 2 rows by 2 columns of cross-tabulation tables stacked in multiple layers (at different periods). If the assumption of equality (Breslow–Day) is rejected, the ORs for each stratum (each period) are considered non-uniform and are adopted. Statistical significance was set at *P* < 0.05.

## Results

Overall, 32,238 SARS-CoV-2 tests were performed (including duplicate tests in the same person); 545 individuals were confirmed to have COVID-19 (283 males, 261 females; median age: 52 years, interquartile range: 33–71 years), and 1487 individuals were close contacts. The number of COVID-19 tests performed was 3360 in period 1, 6368 in period 2, and 22,510 in period 3.

### Characteristics of patients with COVID-19

**Table 2** shows the characteristics of patients with COVID-19 in Fukui Prefecture, Japan. There were 122 patients with COVID-19 in period 1 (median age: 57 years), 122 patients in period 2 (median age: 70 years), and 301 patients in period 3 (median age: 45 years). The number of positive tests differed by patients’ age (*P* < 0.0001): 21–30 years (*P* < 0.001), 51–60 years (*P* < 0.0001), and ≥61 years (*P* < 0.0001). Furthermore, the prevalence of underlying disease differed by period (*P* = 0.005).

**Table 2.** Characteristics of the cases of SARS-CoV-2 infection in Fukui Prefecture, Japan.

	Period 1^*^ (n = 122)	**Period 2^†^ (n = 122)**	**Period 3^‡^ (n = 301)**	***P***
Age (years;IQR)	54 (45.3–66)	59 (42–77)	48 (28–66)	<0.0001^§^
0–10	2 (1.6)	1 (0.8)	12 (4)	0.178
11–20	3 (2.5)	6 (4.9)	24 (8)	0.081
21–30	7 (5.7)	12 (4.9)	55 (18.3)	0.001^||^
31–40	14 (11.5)	10 (8.2)	38 (12.6)	0.444
41–50	18 (14.8)	12 (9.8)	39 (13.0)	0.536
51–60	36 (29.5)	10 (8.2)	43 (14.3)	<0.0001^**^
>61	42 (34.4)	71 (58.2)	90 (29.9)	<0.0001^††^
Sex, n (%)				
Male	74 (60.7)	65 (53.3)	145 (48.2)	–
Female	48 (29.3)	57 (46.7)	156 (51.8)	–
Presence of underlying disease	59 (48.4)	53 (43.4)	96 (31.9)	0.005^‡‡^
Unknown underlying disease	0 (0)	0 (0)	17 (5.6)	–

Fisher's exact test was used for pairwise comparisons of categorical variables. The Kruskal–Wallace test was used for pairwise comparisons of continuous variables. The Holm adjustment was used for multiple comparisons.

* Period 1, March to June 2020;

† Period 2, July to September 2020;

‡ Period 3, October 2020 to February 2021.

§: ^*^ vs. ^†^, *P* < 0.01; ^*^ vs. ^‡^, *P* < 0.01; ^†^ vs. ^‡^, *P* < 0.0001.

|| : ^*^ vs. ^†^, *P* = 0.339; ^*^ vs. ^‡^, *P* = 0.002; ^†^ vs. ^‡^, *P* = 0.077.

**: ^*^ vs. ^†^, *P* < 0.0001; ^*^ vs. ^‡^, *P* < 0.001; ^†^ vs. ^‡^, *P* = 142.

††: ^*^ vs. ^†^, *P* < 0.0001; ^*^ vs. ^‡^, *P* = 0.419; ^†^ vs. ^‡^, *P* < 0.0001. ^††: *^ vs. ^†^, *P* < 0.0001; ^*^ vs. ^‡^, *P* = 0.419; ^†^ vs. ^‡^, *P* < 0.0001.

‡‡: ^*^ vs. ^†^, *P* = 0.521; ^*^ vs. ^‡^, *P* = 0.010; ^†^ vs. ^‡^, *P* = 0.083.

### Characteristics of close contacts

The positivity rates among close contacts are shown in **Table 3**. There were 1487 close contacts: 429 in period 1, 291 in period 2, and 767 in period 3. The number of positive cases among close contacts changed significantly (*P* = 0.009): 57 cases in period 1 (13.3%), 56 cases in period 2 (19.2%), and 154 cases in period 3 (20.0%). There were significant differences between periods 1 and 3 (*P* = 0.010), with period 3 having the most significant proportion of positive cases. In the Cochran–Armitage test, an increasing trend was observed in the infection rate among close contacts (*P* = 0.004).

**Table 3.** Number of close contacts and SARS-CoV-2 positivity rate among close contacts patients with COVID-19 in Fukui Prefecture, Japan.

	Period 1^*^	**Period 2^†^**	**Period 3^‡^**	***P***
Number of close contacts	429	291	767	–
Positivity rate among close contacts, n (%)	57 (13.3)	56 (19.2)	154 (20.0)	0.009

* Period 1, March to June 2020;

† Period 2, July to September 2020;

‡ Period 3, October 2020 to February 2021.

Fisher's exact test was used for pairwise comparisons of categorical variables. The Holm adjustment was used for multiple comparisons. ^*^ vs. ^†^, *P* = 0.073; ^*^ vs. ^‡^, *P* = 0.010; ^†^ vs. ^‡^, *P* = 0.796

Cochran–Amitage test, a test of trend, was used to evaluate the increasing/decreasing trend in the percentage of infections among the close contacts in the first, second, and third periods.

Cochran–Amitage test *P* = 0.004

Stratified independence (Pearson's chi-square) analysis of the COVID-19 test results and the presence or absence of close contacts showed that close contacts were significantly more likely to test positive than other patients tested in all periods (*P* < 0.0001; **Table 4**). As the magnitude of the ORs differed significantly by period (Breslow–Day test and Cochran–Mantel–Haenszel test, *P* < 0.0001), it was not appropriate to calculate a common OR; thus, they were calculated for each period. The ORs for a positive COVID-19 test result among close contacts in each period were as follows: period 1 (OR: 6.76, 95% confidence interval [CI]: 4.66–9.80), period 2 (OR: 21.70, 95% CI: 14.85–31.71), and period 3 (OR: 36.90, 95% CI: 29.04–46.91).

**Table 4.** Odds ratios of having a positive SARs-CoV-2 test result among close contacts of patients with COVID-19.

**Period**	**Close contact with COVID-19 patient**	**SARS-CoV-2 test**		**Stratified independence test (Pearson's χ^2^ test)**	**Stratified odds ratio**	
	
		**Positive**	**Negative**		**Odds ratio**	**95% confidence interval Lower-Upper**
1^*^	No, n (%)	65 (2.2)	2866	*P* < 0.0001	6.76	4.66–9.80
	Yes, n (%)	57 (13.3)	372			
2^†^	No, n (%)	66 (1.1)	6011	*P* < 0.0001	21.70	14.85–31.71
	Yes, n (%)	56 (19.2)	235			
3^‡^	No, n (%)	147 (0.7)	21596	*P* < 0.0001	36.90	29.04–46.91
	Yes, n (%)	154 (20.0)	613			

* Period 1, March to June 2020;

† Period 2, July to September 2020;

‡ Period 3, October 2020 to February 2021.

Odds ratio homogeneity test (Breslow–Day test) *P* < 0.0001. Conditional independence test (Cochran–Mantel–Haenszel correlation statistic) *P* < 0.0001.

Multivariate analysis revealed that female was an independent risk factor of infection in close contacts (Odds ratio; 2.23, 95% CI: 1.700–2.930; **Table 5**).

**Table 5.** Factors that determine the presence or absence of SARS-CoV-2 infection by close contact.

	**Odds ratio**	**95% confidence interval Lower-Upper**	***P***
Age	1.00	0.997–1.010	0.227
Underlying disease	1.19	0.853–1.660	0.306
Female	2.23	1.700–2.930	<0.0001

## Discussion

This study included patients with COVID-19 and their close contacts in Fukui Prefecture, Japan, between March 2020 and February 2021. We compared the definitions of close contacts and SARS-CoV-2 positivity rates in Taiwan, Singapore, China (Guangzhou and Hubei Province), and Japan (Fukui and Nagano Prefectures). We found that the age of patients with COVID-19 significantly decreased; the proportion of patients with underlying disease declined during the study period, probably due to the increase in the proportion of younger patients. The different ORs in the analysis confirming the impact of the stratification (duration) factor also suggest that the infection rate among close contacts changed.

Among COVID-19 close contacts, the elderly and household contacts have higher infection rates, and those with close contact with symptomatic COVID-19 patients reported a 3-fold higher risk of infection than those with close contact with asymptomatic COVID-19 patients [16]. In addition, close contacts who have frequent contact with COVID-19 patients and those exposed to COVID-19 patients with symptoms of coughing are at increased risk of infection [8]. In the present study, women were at risk of being infected by close contacts. The infection rate among close contacts varied greatly depending on the definition of close contacts in each country, and Japan had a high infection rate among Asian countries. Timely quarantine of close contacts is suggested to be necessary for preventing the spread of COVID-19 [17]. The risk of SARS-CoV-2 infection is more frequent among working age women than men [18], which may reflect the higher contact rates among women in general and the fact that women are employed in health care and nursing professions [18]. Presumably, women are the main caretakers of family members with COVID-19 and are more likely than men to be in close contact with the patients, increasing the risk of infection.

The characteristics of age and infection status were as follows: In the first period, infections spread mainly among middle-aged and older people aged ≥40 years. During the first period, a state of emergency was declared by the government, and social restrictions were implemented. For example, restrictions were placed on education institutions, and students were not allowed to attend schools. The low number of COVID-19 cases among those under 20 years of age could be due to the state of emergency. The second period was characterized by infections among people in their 60s or older, possibly due to restaurant-related infections involving karaoke. Karaoke and group singing are popular social activities among the elderly in Japan [19]. Increased risk of SARS-CoV-2 infection was reported when large groups of people participated in karaoke in an enclosed, poorly ventilated room [19, 20]. In the third period, SARS-CoV-2 infection spread mainly among those in their 20s to 50s, largely within families, nursing homes, and companies. Contact tracing in Japan is standardized throughout the country [14]. However, in large cities, it was sometimes difficult to identify close contacts. In Fukui Prefecture, efforts were made to focus on proactive epidemiological investigation, and it was possible to identify the close contacts.

### Infection rates with social distancing, time, and protective measures

Social distancing and the use of masks are significantly associated with the spread of COVID-19 [4]. For preventing SARS-CoV-2 transmission, the analysis shows an apparent synergistic effect among the factors considered (contact time, distance, and use of personal protective equipment [PPE]), which is particularly pronounced when the combination of time factor and use of PPE is considered [21]. In other words, measures that consider contact time, distance, and protective measures are important for preventing infection.

The infection rate due to the difference in the definition of “close contacts” was higher in Fukui Prefecture (period 1) (13.3%) than in Taiwan (0.8%) and China (Guangzhou) (3.7%) [5, 6]. The surveyed periods in Taiwan and China (Guangzhou) partially overlapped with the first period in Fukui Prefecture [5, 6]. When the definition of “close contacts” changed in Japan, the rates were 19.2% in the second period and 20.0% in the third period, which were higher than those in other Asian countries. In Taiwan, the target distance was within 2 m of a patient in a medical facility [5]. Therefore, the Taiwanese infection rates may not take into account the distance from COVID-19 patients outside of medical facilities. The provisions on protective measures covered medical institutions only [5]. We speculate that the infection rate in Taiwan was low because only the contact time was considered in contact tracing of the general population. The definition in China (Guangzhou) specified contact distance (<1 m) and presence or absence of protective measures, but not contact time [6]. The infection rate increased more with the Chinese definition than with the Taiwanese definition. In the Fukui Prefecture (period 1) definition, 3 factors were taken into account: contact time (prolonged), contact distance (<2 m), and protective measures. The infection rate was higher in Fukui Prefecture (13.3%) than in Taiwan (0.8%) and China (Guangzhou) (3.7%) [5, 6, 12]. Although the periods differ, the rates in the second and third periods in Fukui Prefecture were 19.2% and 20.0%, respectively, which were higher when contact time (15 min) and contact distance (<1 m) were more strictly regulated [11]. The attack rate of close contacts is lower than the infection rate but is important in considering the risk of infection among close contacts. The lowest attack rate was 0.7% in Taiwan, 2.4% in Singapore for the contact time and distance (<2 m) factor, and 3.5%–6.5% in China for the contact distance (<1 m) and protective measures factor. The attack rate in Nagano, Japan, was 15.0%, the highest among Asian countries. The survey period was defined by 3 factors: contact time (>15 min), contact distance (<1 m), and the use of masks as protective measures. Contact time, contact distance, and protective measures are the factors associated with infection and attack rates among persons in close contact. In addition, the clarification of contact time of 15 min or more and contact distance of 1 m or less from the change in Japanese definition would further increase the infection rate among close contacts.

Our findings can be used as evidence to educate the population on the importance of social distancing for preventing COVID-19. In addition, differences in the use of masks and other PPE deserve attention. Many previous reports have demonstrated the effectiveness of social distancing and use of masks in preventing COVID-19 [4, 22]. Wearing a face mask lowers infection risk, even in situations with poor social distancing, and social distancing alone is insufficient to prevent the spread of COVID-19 [23, 24]. The time of contact with COVID-19 patients is also important. SARS-CoV-2 is more contagious in indoor environments where particulate matter is suspended for extended durations [25]. Longer residence time in such an environment may increase the risk of SARS-CoV-2 to invade the lower respiratory tract [25].

### Close contacts and contacts of patients with COVID-19

In this study, we needed to understand the definitions of “close contact” and “contact.” The World Health Organization (WHO) guidelines strongly recommend contact tracing to prevent the spread of COVID-19 [26]. The WHO guidelines define “contact” as face-to-face contact with a suspected or confirmed case of COVID-19 within 1 m for >15 min. The definition of “close contact” in Japan references the WHO guidelines and specifies 1 m as the distance to keep from a patient with COVID-19 [11]. Based on the association between positivity rate and distance, contact tracing should be performed for close contacts within a distance of 1 m and contacts within a distance of 2 m. Intensive and effective contact tracing is crucial in preventing the spread of COVID-19.

SARS-CoV-2 mutations continue to occur, and the Omicron variant is now spreading worldwide [27]. SARS-CoV-2 has reached a high prevalence worldwide, with alpha, delta, and omicron variants sequentially spreading across the globe. Omicron is characterized by strong immune escape and is highly contagious, resulting in a global pandemic [28]. A study investigating differences in attack rates of close contacts between the alpha and delta variants of the index COVID-19 patients showed no difference between the 2 variants [29]. By contrast, the attack rate of unvaccinated close contacts was higher for the delta variant than for the alpha variant [29]. The attack rate among close contacts suggests that vaccination is involved [29]. In other words, the infection and attack rates among close contacts are likely to be influenced by vaccination. Thus, it is important to improve vaccination coverage and contact tracing and be stricter regarding social distancing and the use of masks in group settings.

These include the presence or absence of preventive measures, higher rates during epidemics when there are many infected persons, and lower rates during periods of natural infection or when immunity from vaccination has taken hold of close contacts.

This study has a few limitations. First, some individuals were tested more than once; thus, the total number of SARS-CoV-2 tests performed is greater than the number of individuals tested. Second, the characteristics of people who tested negative by the PCR test are unknown. Third, the details regarding underlying diseases are unknown. Fourth, since the study period, newer viral variants and subvariants have emerged (such as Omicron), and these were not included in the study. Fifth, the cross-sectional design cannot make a causal inference of positive cases and close contact. Therefore, the factors mentioned earlier should be considered in future studies.

## Conclusions

In the present study, female sex was a risk factor for transmission by close contacts. Our findings also suggested that the rate of infection among close contacts was associated with contact duration, contact distance, and protective measures. Thus, the infection rate of close contacts varies depending on many factors.
